# Interactions between *Candida albicans* and the resident microbiota

**DOI:** 10.3389/fmicb.2022.930495

**Published:** 2022-09-20

**Authors:** Hao Li, Ming-xing Miao, Cheng-lin Jia, Yong-bing Cao, Tian-hua Yan, Yuan-ying Jiang, Feng Yang

**Affiliations:** ^1^Department of Pharmacy, Shanghai Tenth People’s Hospital, Tongji University School of Medicine, Shanghai, China; ^2^Department of Physiology and Pharmacology, School of Basic Medicine and Clinical Pharmacy, China Pharmaceutical University, Nanjing, China; ^3^Institute of Vascular Disease, Shanghai TCM-Integrated Hospital, Shanghai University of Traditional Chinese Medicine, Shanghai, China

**Keywords:** *Candida albicans*, microbiota, biofilm, candidiasis, polymicrobial disease

## Abstract

*Candida albicans* is a prevalent, opportunistic human fungal pathogen. It usually dwells in the human body as a commensal, however, once in its pathogenic state, it causes diseases ranging from debilitating superficial to life-threatening systemic infections. The switch from harmless colonizer to virulent pathogen is, in most cases, due to perturbation of the fungus-host-microbiota interplay. In this review, we focused on the interactions between *C. albicans* and the host microbiota in the mouth, gut, blood, and vagina. We also highlighted important future research directions. We expect that the evaluation of these interplays will help better our understanding of the etiology of fungal infections and shed new light on the therapeutic approaches.

## Introduction

*Candida* species are commensal colonizers of the human body. They dwell in several niches including the oral cavity, gastrointestinal tract, and vagina. Up to 60% of healthy individuals are colonized by *Candida* spp. (Kullberg and Arendrup, 2015; McCarty and Pappas, 2016). *Candida* spp. are also the most prevalent opportunistic human fungal pathogens. They can cause superficial and dermal infections as well as life-threatening invasive candidiasis (Pappas et al., 2018). They are the fourth most common cause of nosocomial bloodstream infections and are the major fungal pathogens isolated from medical device infections (Wisplinghoff et al., 2004; Pfaller and Diekema, 2007). Among the approximately 200 species in the genus *Candida*, at least 30 of them can cause human disease (Brandt and Lockhart, 2012), but most of *Candida*-related infections are caused by *C. albicans*, followed by other non-albicans *Candida* species, including *C. glabrata*, *C. tropicalis*, *C. parapsilosis*, and *C. krusei* (Pfaller et al., 2019).

In the host, many microorganisms exist as biofilms. Thus, *C. albicans* usually exists as a heterogeneous biofilm with microorganisms in the host (Diaz et al., 2014), and infections caused by *C. albicans* are usually (30–60%) polymicrobial diseases (Sheehan et al., 2020). Under normal conditions, the host’s innate and acquired defense mechanisms and the resident microbiota act in concert to ensure *C. albicans* inhabits as a commensal. Innate and acquired immune response to *C. albicans* infections have been extensively reviewed previously (Netea et al., 2008; Richardson and Moyes, 2015; Qin et al., 2016; Pellon et al., 2020). Trillions of microorganisms reside in and on the human body. Some microorganisms are protective to the host while some could be potential pathogens. Under normal circumstances in healthy hosts, the microbiota has a balanced composition of metabolites and energy. The homeostatic equilibrium prevents the overgrowth of potentially pathogenic microorganisms. Imbalance of the microbiota community, also known as dysbiosis, is often associated with human diseases (DeGruttola et al., 2016; El-Sayed et al., 2021; Mizutani et al., 2022). In this review we summarize *in vitro* and *in vivo* interactions of *C. albicans* with the resident microbiota, as well as the molecular mechanisms. We also highlighted some questions that have been previously ignored and discuss suggestions for future research.

## Interactions between *C. albicans* and microbiota in the oral cavity

Human oral cavity provides multiple habitats for the microbiota, including teeth, tongue, cheek, gingival sulcus, attached gingiva, lip, hard, and soft palates. Over 700 different species of bacteria, together with other microorganisms such as fungi, viruses and protozoa, live in the human oral cavity (Marsh and Zaura, 2017). *Candida* spp. are the most frequent fungi isolated from the oral cavity (Byadarahally Raju and Rajappa, 2011). Oral candidiasis, also known as oral thrush, is a common opportunistic fungal infection of the oral cavity, especially in the elderly or hospitalized patients (Scully et al., 1994; Abu-Elteen and Abu-Alteen, 1998; Fanello et al., 2006). It is mostly caused by overgrowth of *C. albicans* (Millsop and Fazel, 2016; Hellstein and Marek, 2019). The annual global incidence of oral candidiasis is approximately 2 million (Dufresne et al., 2017). Among HIV-infected patients, oropharyngeal candidiasis (OPC) is the most common oral fungal infections. More than 90% of HIV-positive patients develop OPC at some stage of their disease (Flint et al., 2006).

## Bidirectional interactions between *C. albicans* and oral bacteria

Some oral bacteria enhance while others inhibit biofilm formation in *C. albicans*. In an *in vitro* measurement of mixed species biofilm formed on the salivary pellicle, *Streptococcus* spp. and *Actinomyces* spp. up-regulated the expression of *C. albicans* virulence genes and hyphal and biofilm development of *C. albicans*. *Streptococcus oralis* also enhanced hyphal and biofilm development of *C. albicans* (Cavalcanti et al., 2017). In another study, when co-cultured with *C. albicans* on denture acrylic, *Porphyromonas gingivalis* inhibited expression of virulence genes and production of hyphae of *C. albicans* (Morse et al., 2019). In contrast, a mouse intravenous chemotherapy model, *C. albicans* infection of 5-fluorouracil-immunosuppressed mice caused an overgrowth but decreased diversity of oral bacteria (Bertolini et al., 2019). A study measured 82 Dutch adults for the relative abundance of salivary *Candida* and bacteria. The study revealed a negative correlation between *Candida* load and the bacterial profiles of human saliva: increased *Candida* load was accompanied by decreased bacterial diversity (Kraneveld et al., 2012).

## Interactions between *C. albicans* and *S. gordonii*

*Streptococcus gordonii* is one of the first colonizers of the oral cavity (Frandsen et al., 1991). In an *in vitro* mucosal model, *S. gordonii* increased the mucosal invasion of the co-cultured *C. albicans* (Diaz et al., 2012). In *C. albicans*, deletion of *EFG1* and *BRG1* caused defection of adhesion and hyphae formation. The co-cultured *S. gordonii* could restore the ability of forming biofilms in these deletion strains (Montelongo-Jauregui et al., 2019). In the mixed species biofilms formed by *C. albicans* and *S. gordonii*, the extracellular matrix produced by *C. albicans* conferred resistance to antifungal drugs and antibiotics (Montelongo-Jauregui et al., 2016, 2018, 2019). In an *in vitro* test, *S. gordonii* and *C. albicans* were co-cultured on the surface of 24-well culture plates. *S. gordonii* increased the production of host cytokines IL-8 and TNF-α *via* up-regulating the expression of the cytokine genes (Bhardwaj et al., 2020). When co-infected with *S, gordonii*, *C. albicans* could survive more in murine macrophages, and escape more from murine macrophages, and form larger size of microcolonies on murine macrophage monolayers (Salvatori et al., 2020). In the *in vitro* mucosal model mentioned above, *C. albicans* promoted biofilm formation of *S. gordonii* (Diaz et al., 2012).

Physical contact and chemical signals are involved in the interactions between *C. albicans* and *S. gordonii*. The physical contact is mediated by the hyphal-wall proteins Als3p, Eap1p, and to a lesser degree Hwp1p of *C. albicans*, and *S. gordonii* cell wall-anchored proteins SspA and SspB of *S. gordonii*. The chemical interactions involve the production of autoinducer 2 by *S. gordonii*, which induces hyphal formation, and suppression of farnesol (the quorum sensing molecule of *C. albicans*) inhibition of hyphal formation *of C. albicans* (Holmes et al., 1998; Bamford et al., 2009; Nobbs et al., 2010; Silverman et al., 2010). The N-terminal domain of Als3p is required for the physical contact (Bamford et al., 2015). The early O-mannosylation is a critical stage required for activation of hyphal adhesin functions, recognition by *S. gordonii* and mixed species biofilm formation (Dutton et al., 2014). The only *S. gordonii* glucosyltransferases (Gtf) enzyme, GtfG, promotes coaggregation with *C. albicans*, binding to *C. albicans* preformed biofilms, and formation of mixed biofilm (Ricker et al., 2014). Similarly, the only glucosyltransferases (Gtf) enzyme of *S. oralis*, GtfR, also enhances mixed species biofilm formation by *S. oralis* and *C. albicans* (Souza et al., 2020). The comCDE operon (Jack et al., 2015) and the fruRBA operon (Jesionowski et al., 2016) are required for the development of enhanced mixed species biofilm. Compared to wild type *C. albicans* strain, co-culturing of *S. gordonii* with *C. albicans* strains with deletions of *LEU3*, *CAS4*, *CTA4*, and *SKO1* resulted in heavier biofilm but coculturing of *S. gordonii* with *C. albicans* strains with deletions of *SFL2*, *BRG1*, *TEC1*, *TUP1*, *EFG1*, and *RIM101* resulted in lighter biofilms. Co-culturing of *S. gordonii* with *C. albicans* strains with deletions of *SFL2*, *TEC1*, and *EFG1* also caused reduced tolerance to antibiotics (Chinnici et al., 2019).

## Interactions between *C. albicans* and *Streptococcus mutans*

*Streptococcus mutans* is the major species associated with dental caries (Loesche, 1986). *S. mutans* is often isolated together with *C. albicans* in early childhood caries (Raja et al., 2010; Qiu et al., 2015a,b). In mixed biofilm formed by *C. albicans* and *S. mutans* on poly-L-lysine-coated glass slides, *S. mutans* secreted glucotransferase B (GtfB), which bound to the surfaces of *S. mutans* and *C. albicans*. GtfB bound to the surface of *C. albicans* produced more glucan than GtfB bound to the surface of *S. mutans* (Hwang et al., 2015). The glucans are binding sites for *S. mutans* (Gregoire et al., 2011) and the glucans enhance *S. mutans* adhesion and cohesion to the surface of teeth (Bowen and Koo, 2011). GtfB itself increases *C. albicans* biofilm formation, even in the *bcr1*Δ/Δ strain by up-regulating the expression of *HWP1*, *ALS1*, and *ALS3* in wild type and *bcr1*Δ/Δ strains of *C. albicans* (Ellepola et al., 2017). In an *in vivo* dental plaque biofilm model using healthy female rats, the mannans provided by *C. albicans* in the outer surface of the cell wall acted as the binding sites of GtfB, which increased the production of the glucan-matrix and modulated the interspecies interaction in the dual species biofilm (Hwang et al., 2017). The secreted polysaccharides by *C. albicans* might also contribute to the dual species biofilm formation *in vitro* in 96-well plates and *in vivo* in immunosuppressed mice (Khoury et al., 2020).

In dual species biofilms formed in 96 well plates, GtfB directly enhanced antifungal tolerance of *C. albicans* to fluconazole *via* the exopolysaccharides (EPS) matrix, which were mostly glucans synthesized by GtfB and GtfC. EPS directly bound and sequestered fluconazole. GtfB indirectly enhanced antifungal tolerance of *via* increasing the expression of *C. albicans* genes involved in biofilm formation (Ellepola et al., 2017). In an *in vitro* biofilm model using hydroxyapatite discs coated with saliva, inhibition of GtfB activity by povidone iodine re-established *C. albicans* susceptibility to fluconazole (Kim et al., 2018). *C. albicans* also plays an active role by up-regulating genes involved in carbohydrate metabolism, cell morphogenesis and cell wall components such as mannan. GtfB can enhance *C. albicans* growth and acid production *via* breaking down sucrose into glucose and fructose, which can be readily metabolized by *C. albicans*. Environmental acidification is a key virulence factor associated with the onset of caries (Ellepola et al., 2019). In dual species biofilms formed on hydroxyapatite discs coated with saliva, *C. albicans* could augment the production of exopolysaccharides, which was a key mediator of mixed species biofilm development and induce the expression of *S. mutans* virulence genes, including *gtfB*. As a result, the dual species biofilms had more biomass and more viable *S. mutans* cells than single-species biofilms (Falsetta et al., 2014). In an *in vivo* murine dental caries model, compared to rats infected with *C. albicans* or *S. mutans* alone, co-infected rats displayed higher levels of infection and had more microbial abundance within plaque biofilms. Co-infection also caused higher virulence of plaque biofilms in rats than single-species infection, indicated by the level of colonization by *S. mutans* and *C. albicans* in the plaque biofilm, and the onset of carious lesions on teeth surface (Falsetta et al., 2014). On the other hand, when co-cultured in 96-well plates, *S. mutans* secreted some products which were capable of inhibiting *C. albicans* morphogenesis, biofilm formation, and pathogenicity (Barbosa et al., 2016; Dos Santos et al., 2020). Co-culturing of *C. albicans* and *S. mutans* on glass microscope slides, farnesol inhibited formation of single and mixed species biofilms (Koo et al., 2003; Fernandes et al., 2016). However, in another study, using an *in vitro* model of biofilms formed on hydroxyapatite discs coated with saliva, low concentrations of farnesol enhanced *S. mutans* biofilm formation and GtfB activity (Kim et al., 2017).

In addition to streptococci, the periodontal pathogens *Aggregatibacter actinomycetemcomitans* and *Fusobacterium nucleatum* also interact with *C. albicans*. *In vivo*, they could excrete the quorum sensing molecule AI-2, which inhibits germination of *C. albicans* (Bachtiar et al., 2014; Bor et al., 2016).

## Interactions of *C. albicans* and microbiota in the gut

As a commensal dweller of the human gastrointestinal (GI) tract, upon disruption to the host immune system or imbalance of the microbiota, *C. albicans* can disseminate from the GI tract and cause systemic infections with high mortality (d’Enfert et al., 2021). Within the gut, multiple factors can influence the diversity and density of the microbiota, including environmental parameters such as abundance of oxygen and nutrients, and pH (Donaldson et al., 2016). Abundance of *C. albicans* is also regulated by the gut microbiota and vice versa. For example, *Firmicutes* and *Bacteroides* restrict colonization of *C. albicans* in the mouse gut by activating transcription factor HIF-1α in intestinal cells, which causes an increase in the antimicrobial peptide LL-37 (Fan et al., 2015). *C. albicans* and lactic acid bacteria (LAB) have the same metabolic niches throughout the GI tract. Dysbiosis of *C. albicans* causes altered levels of LAB, especially *Lactobacillus* spp. and *Enterococcus* spp. Lactobacilli inhibit *C. albicans*, while *Enterococcus faecalis* and *C. albicans* are mutualistic (Zeise et al., 2021). In an *in vitro* gut model, *Lactobacillus rhamnosus* reduced *C. albicans* hyphal elongation, drove the shedding of hyphae from the surface of epithelial cells and protected against *C. albicans* induced epithelial damage (Graf et al., 2019).

## Effect of broad-spectrum antibiotics on *C. albicans*

Abuse of broad-spectrum antibiotic treatment depletes gut bacteria and facilitates fungal overgrowth through fungal dysbiosis and increases the risk of disseminated candidiasis (Seelig, 1966a,b; Mason et al., 2012; Dollive et al., 2013). In immunocompromised patients, broad-spectrum antibiotics induced imbalance in the normal bacterial flora is a prerequisite for colonization of *C. albicans* (Högenauer et al., 1998). Broad-spectrum antibiotics cause overgrowth of *C. albicans* directly and indirectly. The direct effect is due to the killing of a large proportion of the microbiota, and the indirect effect is due to decreased colonization resistance within the GI-tract (van Ogtrop et al., 1991). Colonization resistance is a term used to describe the capacity of the healthy microbiota to limit the introduction and/or expansion of potential pathogens (Khan et al., 2021). Altered microbiota caused by antibiotic treatment can persist for months, thereby causing long-term decreases in beneficial anaerobic organisms (Sjövall et al., 1986; Lidbeck and Nord, 1993; Orrhage and Nord, 2000), and increases in potential pathogens (Guggenbichler et al., 1985; van der Waaij, 1989; Samonis et al., 1993; Payne et al., 2003). One of the most common side effects of broad-spectrum antibiotic treatment is yeast infections at mucosal sites, including those caused by *C. albicans* (Giuliano et al., 1987; Samonis et al., 1993; Sullivan et al., 2001).

In addition to directly promoting the overgrowth of *C. albicans*, antibiotics can also directly alter fungal metabolism. The β-lactam antibiotics, such as cefepime, amoxicillin, and vancomycin, can stimulate the *in vitro* planktonic growth of *C. albicans* in 96-well plates and *in vivo* virulence tested with *Caenorhabditis elegans* infection model (Aguiar Cordeiro et al., 2018). Cefepime and amoxicillin also directly stimulate *C. albicans* metabolism and biofilm formation in 96-well plates, thereby enhancing antifungal tolerance (Cordeiro et al., 2019). Gut bacteria treated with β-lactam antibiotics released significant amounts of peptidoglycan fragments, a procedure so called “a peptidoglycan storm” (Tan et al., 2021), which strongly stimulated hyphal formation in *C. albicans in vitro* and *in vivo* (Xu et al., 2008; Tan et al., 2021). Hyphal growth is a key virulence factor required for penetration of mucosal barriers (Lo et al., 1997). The altered gut metabolites caused by antibiotic treatment include primary and secondary bile acids. The primary bile acid taurocholic acid promoted growth and induces the expression of hyphae-specific genes *ECE1*, *UME6*, *HWP1*, and *HCG1*, thereby enhancing hyphal formation of *C. albicans in vitro* (Hsieh et al., 2017; Guinan and Thangamani, 2018). In contrast, the secondary bile acids (lithocholic acid and deoxycholic acid) inhibited *C. albicans* growth, adherence, hyphae and biofilm formation *in vitro* in 96-well plates (Guinan et al., 2018). Cefoperazone treatment caused an increased level of the primary bile acid taurocholic acid in mice gut. In addition, cefoperazone treatment changed the microbiome at both phyla-level and family level in mice gut. It caused a higher abundance of *Firmicutes* and lower abundance of *Bacteroidetes* and *Verrucomicrobia*, a higher abundance of *Panibacillaceae* and lower abundance of *Lactobacillaceae*, *Turicibacteraceae*, *Clostridiales*, and *Clostridiaceae*. Cefoperazone treatment also altered the ability of *C. albicans* to modify the mice gut bacterial microbiota (Gutierrez et al., 2020).

## Relationship between overgrowth of *C. albicans* and diseases

Overgrowth of *C. albicans* in the gut may lead to health problems, such as digestive problems (gas and diarrhea), and psychological disorders (anxiety, memory loss, depression) (Severance et al., 2016; Markey et al., 2020). In high-risk patients, *C. albicans* might translocate from the GI tract to extra-intestinal organs, especially the liver, the spleen, and the bloodstream. The strain causing systemic candidiasis and the strain identified from the same patient’s rectum sample are always the same, although in some cases, catheters are another source of infection (Miranda et al., 2009). *C. albicans* in the GI tract may also be an immunogen which triggers or promotes a variety of hypersensitivity diseases (Goldman and Huffnagle, 2009). However, the direct evidence of *C. albicans* associated diseases is still lacking (Li et al., 2018b,a; Kapitan et al., 2019). Reduced growth of *C. albicans* can also cause diseases. In mice, reduced growth of *C. albicans* from treatment with the antifungal drug fluconazole increased the immune response and severity of experimentally induced colitis and induced allergic airway disease (Wheeler et al., 2016; Li et al., 2018b).

## Interaction of *C. albicans* with bacteria in the blood

Invasive candidiasis is a global health threat with high morbidity and mortality, as well as costly and lengthy hospital stays (Morgan et al., 2005; Pfaller and Diekema, 2007; Horn et al., 2009; Wu et al., 2017). Candidemia is the most common form of invasive candidiasis (Magill et al., 2014, 2018; Ricotta et al., 2020). *C. albicans* is the most frequent fungal pathogen and *Staphylococcus* spp. are the most frequent bacterial pathogens isolated from blood stream infections worldwide. In approximately 20% of all *C. albicans* bloodstream infections, *C. albicans* was co-isolated with *Staphylococcus epidermidis* or *Staphylococcus aureus* (Klotz et al., 2007; O’Donnell et al., 2015; Reno et al., 2015).

*Staphylococcus epidermidis* co-cultured in polystyrene tubes with *C. albicans* enhanced adhesion and biofilm formation of *C. albicans* (El-Azizi et al., 2004). Formed on optical microwell Petri dishes, the dual species biofilms of *C. albicans* and *S. epidermidis* were sicker than single species biofilms and had more dissemination of *S. epidermidis in vivo* in mouse model of subcutaneous catheter biofilm infection (Pammi et al., 2013).

Physical interactions of *C. albicans* with *S. aureus* and *S. epidermidis* involve *C. albicans* hyphae protein Als1p, Als3p, and cell surface O-linked mannosylations. Als3p, which is expressed exclusively in the hyphae, is the receptor for binding of *S. aureus* and *S. epidermidis* (Peters et al., 2010, 2012; Beaussart et al., 2013). Als1p and cell surface O-linked mannosylations can bind specific peptide ligands on surface of *S. epidermidis* (Beaussart et al., 2013). The physical interaction is required for bacterial invasion. The association of *S. aureus* with *ALS3*—expressing *C. albicans* in the oral cavity resulted in *S. aureus* bacteremia and the isolation of *S. aureus* bacteria from kidney tissue (Schlecht et al., 2015). *C. albicans* hyphae is highly immunogenic (Ballou et al., 2016). It attracts phagocytic cells, which rapidly engulf the adherent *S. aureus*, and subsequently migrate to cervical lymph nodes, from where *S. aureus* disseminate into the internal organs and cause systemic infections (Allison et al., 2019). Both organisms benefit from this interaction, which modulates the host immune response differently than monospecies infections (Peters and Noverr, 2013). In a *Galleria mellonella* larvae disseminated infection model, co-infection with *C. albicans* and *S. aureus* resulted in a reduced survival rate compared to a monospecies infection (Sheehan et al., 2020). In the murine model of intra-abdominal infection, *C. albicans* raised the pH during polymicrobial growth. The elevated pH drove the expression of the accessory gene regulator quorum sensing system, thereby increased alpha-toxin production. Thus, coinfection of *C. albicans* and *S. aureus* enhanced the virulence of *S. aureus* (Todd et al., 2019a,b).

Farnesol prevents biofilm formation of *C. albicans* on the surface of microtiter plates, and it prevents biofilm formation of *S. aureus* in polystyrene plates (Ramage et al., 2002; Jabra-Rizk et al., 2006). But the effect of farnesol on biofilm formation of *S. aureus* is dose-dependent: at low concentration, farnesol increases biofilm formation, but at high concentration, it inhibits biofilm formation. Whereas tyrosol, another quorum sensing molecule of *C. albicans*, has no effect on *S. aureus* biofilm formation in 12-well plates (Krause et al., 2015). However, prostaglandin E2, produced by *C. albicans* from external arachidonic acid, stimulated *S. aureus* biofilm formation in the mixed biofilm in 12-well plates (Krause et al., 2015). In addition, in the mixed biofilm formed in a mouse biofilm infection model using subcutaneous catheter, the bacterial repressor of autolysis (the lrg operon) was down-regulated, thereby the bacterial autolysis was enhanced. Bacterial autolysis is a procedure of self-digestion of the cell wall mediated by the peptidoglycan hydrolase autolysins (Lewis, 2000). The down-regulation might be the cause of enhanced extracellular DNA (eDNA) levels in this condition (Pammi et al., 2013). Autolysis of *S. epidermidis* results in release of eDNA into the extracellular matrix. In addition, *C. albicans* can actively secrete eDNA. eDNA could enhance adhesion and biofilm formation (Pammi et al., 2013). Furthermore, in the mixed biofilm formed in 96-well flat-bottom plates, *C. albicans* could enhance the pathogenicity of *S. aureus* through increasing tolerance to antimicrobials. Farnesol induced bursts of reactive oxygen species, which triggered the expression of efflux pumps in *S. aureus* (Kong et al., 2017; Vila et al., 2019). In addition, in the mixed biofilm matric, *C. albicans* could secrete β-1,3-glucan, which prevented penetration of drugs and enhances tolerance to antimicrobial drugs (Kong et al., 2016).

## Interactions between *C. albicans* and microbiota in the vagina

Many females suffer from vulvovaginal infections (VVI). Approximately 75% of women develop VVC at least once in their lifetime (Sobel, 2007). Mixed vaginal infections represent >20% of women with VVI (Deidda et al., 2016). Bacterial Vaginosis (BV), Vulvovaginal Candidiasis (VVC) and Trichomoniasis (TV) are the three most prevalent VVI (Kalia et al., 2017, 2018, 2019). Recurrent vulvovaginal candidiasis (RVVC) afflicts about 8% of women globally (Denning et al., 2018). VVC is usually caused by the overgrowth of the *Candida* spp. (De Gregorio et al., 2020). Until recently, *C. albicans* was the species most detected in VVC. However, during the last two decades, non-albicans *Candida* (NAC) species have emerged increasingly as causative species of VVC (Rodríguez-Cerdeira et al., 2019).

*Candida* is a commensal dweller of the genitourinary tract with colonization rates of 11.6–17% (Andrioli et al., 2009). The composition of the vaginal microbiota is dynamic and is subject to regulation by physical conditions such as menstruation, pregnancy, and health status (Greenbaum et al., 2019). However, multiple factors can cause fungal overgrowth in the vagina. These factors include wide-spectrum antibiotic use, immunosuppressive therapy, and alterations of the vaginal conditions due to, for example, changes of nutrients or fluctuations of pH (Nobile and Johnson, 2015; Romo and Kumamoto, 2020). As an opportunistic pathogen, *C. albicans* can cause cutaneous and mucocutaneous candidiasis inside the vaginal. Once disseminated into inner organs or the bloodstream, *C. albicans* can also cause life-threatening invasive infections (Hube, 2004; Hall and Noverr, 2017).

## Interactions between *C. albicans* and group B *Streptococcus*

Group B *Streptococcus* (GBS) is usually found in human rectum and vagina. It is a leading cause of neonatal sepsis, pneumonia, and meningitis worldwide and is the leading cause of death among newborns in developed countries. GBS can be vertically transmitted from mother to neonate. Therefore, maternal vaginal colonization is a key risk factor for neonatal disease caused by GBS (Chen et al., 2018). *In vitro* data indicated that, the GBS antigen I/II family adhesins directly bind to the vaginal epithelium (Rego et al., 2016), and directly bind to Als3p of *C. albicans*, thereby enhancing association of *C. albicans* with vaginal epithelium (Pidwill et al., 2018).

## Interactions between *C. albicans* and lactobacilli

*Lactobacillus* spp. are the major bacteria in the vaginal microbiota of healthy women. *Lactobacillus* spp. prevents the vagina from urogenital and sexually transmitted infections *via* secreting metabolites such as organic acids, hydrogen peroxide (H_2_O_2_), bacteriocins, and biosurfactants (Nader-Macías and Juárez Tomás, 2015; Mendling, 2016; Wang et al., 2017; Fuochi et al., 2019; Linhares et al., 2019). Organic acids secreted by *Lactobacillus* spp. are the major cause of acidic pH (4–4.5) in the vaginal environment, and are the inhibitive factor of pathogen growth (Miller et al., 2016). H_2_O_2_-generating *Lactobacillus* spp. are in the vagina of approximately 96% of healthy women, while their populations are lower in women with VVI (Boris and Barbés, 2000). Some bacteria, such as *L. fermentum* (Pascual et al., 2008), *Streptococcus sanguinis* (Ma et al., 2015), *E. faecalis* (Graham et al., 2017; De Cesare et al., 2021) are capable of producing bacteriocins that inhibit growth, yeast-to-hyphae switch, biofilm formation, and virulence of *C. albicans*. Bacteriocins are antimicrobial peptides or proteins produced by bacteria (Benítez-Chao et al., 2021). Biosurfactants are surface-active biomolecules produced by microorganisms (Healy et al., 1996). Biosurfactants are important mediators of cell adherence and desorption from surfaces (Desai and Banat, 1997). *Lactobacillus*-derived biosurfactants can alter the hydrophobicity and electrical properties of interfaces, thereby inhibiting fungal adhesion and biofilm formation (De Gregorio et al., 2020; Zeise et al., 2021).

*Lactobacillus crispatus* is the dominant *Lactobacillus* spp. in healthy vagina (Ceccarani et al., 2019). *In vitro* tests indicated the organic acids and H_2_O_2_ produced by *L. crispatus* can strongly inhibit *C. albicans* growth and hyphal formation. In addition to lactate, *in vitro* tests indicated *L. crispatus* also produced other anti-*Candida* small molecules, which inhibited growth and hyphal morphogenesis of *C. albicans*. *L. crispatus* also down-regulated the expression of *ALS3*, *HWP1*, and *ECE1*, thereby limiting the virulence of *C. albicans* (Wang et al., 2017). Resistance to VVC increased when *L. crispatus* was the dominant bacteria community in the vaginal environment (Jang et al., 2019). *L. casei* also has strong anti-*Candida* effect. *L. casei* co-cultured in 96-well flat-bottom polystyrene plates with *C. albicans* or *C. tropicalis* reduced the formation of fungal hyphae and early biofilms (Sobel, 2016; Paniágua et al., 2021). In contrast, *in vitro, L. iners* up-regulated expression of *HWP1* and *ECE1*, induced hyphal growth and increased both the biofilm biomass and metabolic activity of *C. albicans*. Furthermore, *L. iners* can increase C. albicans virulence *via* transforming *C. albicans* isolates with moderate or weak biofilm-forming ability to strong biofilm producers (Sabbatini et al., 2021).

*Lactobacillus* spp. also inhibits *C. albicans* by competing for adhesion sites *in vitro* (Allonsius et al., 2017). *C. albicans* invades vaginal epithelial cells mainly through endocytosis and active penetration. *Lactobacillus* spp. such as *L. crispatus* and *L. plantarum* can specifically adhere to the vaginal tissue, form biofilm and inhibit adhesion of *C. albicans* by several mechanisms, including competition for receptors, displacement of adhered pathogenic microorganisms, and avoiding their re-adhesion (Boris et al., 1998; Coudeyras et al., 2008; Borges et al., 2014; De Seta et al., 2014).

## Future directions

In addition to the above-mentioned interplays in the microbiota, we think the following directions deserve further investigations in the future.

## Effect of anti-fungal use on the microbiota

Many antibiotics can disrupt the microbiota equilibrium and eliminate “good” gut bacteria which inhibit fungal growth. Therefore, abuse of antibiotics promotes fungal overgrowth and pathogenicity. Few studies have looked at the effect of anti-fungal use on the microbiota. Some studies indicate fungal depletion due to application of antifungal drugs is associated with altered microbiome and some diseases. For example, in one study, mice treated with antifungal drug fluconazole had severe dextran sulfate sodium-induced colitis (DSS-colitis), while mice treated with antibiotics had less severe DSS-colitis. One hypothesis was fluconazole caused the deletion of commensal fungi in the gut. The depletion led to overgrowth of pathogenic bacteria, which exacerbated colitis (Qiu et al., 2015b). Similarly, in another study, treatment of mice with fluconazole caused more aggravated DSS-induced acute colitis, as well as T cell transfer-mediated chronic colitis, and house dust mite-induced intratracheal allergic airway disease (Iliev et al., 2012; Qiu et al., 2015b; Wheeler et al., 2016). Further investigations indicated that fluconazole caused dysbiosis of both mycobiota and bacteriobiota. Among the mycobiota, *Candida* spp. were decreased but other fungi, such as *Aspergillus*, *Wallemia*, and *Epicoccum*, were increased. Among the bacteriobiota, the overall diversity remained unchanged, but *Bacteroides*, *Allobaculum*, *Clostridium*, *Desulfovibrio*, and *Lactobacillus* spp. had decreased relative detection, while *Anaerostipes*, *Coprococcus*, and *Streptococcus* had increased relative detection (Wheeler et al., 2016)

## Therapy against mixed biofilms

Both singularly or co-existed, fungi and bacteria usually form biofilms in the human body. Forming of mixed species biofilms protects both bacteria and fungi from external assaults, such as host clearance and antimicrobial agents, and facilitates intra- and inter-species interactions (Levison and Pitsakis, 1987; Shirtliff et al., 2009). For example, it was found that mixed species biofilms of *C. albicans* and oral streptococci were more resistant to antibiotics than single species biofilms (Morales and Hogan, 2010). The most common mixed fungal infections are caused by mixing of *C. albicans* with *S. aureus*, *P. aeruginosa* or *Escherichia coli* (Dhamgaye et al., 2016; Kalan and Grice, 2018). Mixed infections of two or three *Candida* spp. are also found in patients with orogastric cancer (de Sousa et al., 2016). The overall prevalence of fungal mixed fungal infections almost doubled over the 10-year period, with 10.3% of all infections in 2015 compared to 5.7% in 2006 (Gawaz and Weisel, 2018). However, traditional therapies against microbial infections generally target individual causative agents. Considerations for efficacy on a polymicrobial cause or on individual members of microbial communities are largely lacking. Treatment of mixed species infections should be the combinations of antimicrobials, and the drugs of choice should be synergistic. Potential antagonism between drugs and undesirable side effects of the drugs also needs to be taken into consideration.

## Restoring microbiota equilibrium

In addition to candidiasis, imbalanced microbiota has been linked directly and indirectly to various human diseases including mental illnesses (Clapp et al., 2017), autoimmune diseases (Xu et al., 2019), and chronic diseases such as rheumatoid arthritis and asthma (Vijay and Valdes, 2022). Numerous approaches have been developed with the purpose of rehabilitating the perturbed microbiota, such as the administration of probiotics, prebiotics, and synbiotics, fecal microbiota transplantation, phages, predatory bacteria (Gagliardi et al., 2018; Gomaa, 2020; Sestito et al., 2020).

Probiotics can be used to prevent the onset of dysbiosis and to restore the balance in the dysbiosis (Gomaa, 2020). Vaginal administration of probiotics is a promising therapy of VVC. In VVC patients, probiotics could potentiate the efficiency of azoles (Kovachev and Vatcheva-Dobrevska, 2015). *L. acidophilus*, *L. rhamnosus*, and *L. reuteri* are the most-researched strains of probiotics for the purpose of restoring and maintaining vaginal microbiota equilibrium (Reid et al., 2001, 2003). Probiotics can stick to vaginal surfaces and inhibit growth of harmful bacteria on vaginal surfaces. *Lactobacillus* may also adhere directly to harmful bacteria and prevent them from spreading (Maudsdotter et al., 2011). In addition, *L. rhamnosus* GG can interfere with hyphal formation of *C. albicans via* production of exopolysaccharides and chitinases (Allonsius et al., 2017, 2019). In addition to probiotic bacteria, some yeasts are also potential probiotics. For example, both live and inactivated *Saccharomyces cerevisiae* can co-aggregate with *C. albicans* and inhibit adherence of *C. albicans* to epithelial cells. *S. cerevisiae* can also inhibit the switch from yeast to mycelial form, production of aspartyl proteases (SAP) in *C. albicans* (Pericolini et al., 2017). Another well characterized probiotic yeast, *Saccharomyces boulardii*, is often used to alleviate GI tract disorders. It also inhibits adhesion of *C. albicans* to and biofilm formation on polystyrene surfaces (Krasowska et al., 2009).

Fecal microbiota transplantation (FMT) can restore the compositions and functions of gut microbiota. It has been successfully used for the treatment of several intestinal diseases, such as *Clostridium difficile* infection, ulcerative colitis, and Crohn’s disease (Tan et al., 2020). Autologous FMT has the potential of restoring a pre-antibiotic microbiome baseline after disruption (Bulow et al., 2018; Suez et al., 2018; Taur et al., 2018). The presence of *C. albicans* in the donor stool decreases the efficacy of FMT, suggesting the outcome of FMT is largely affected by fungal dysbiosis (Zuo et al., 2018). FMT of commensal bacteria prevents *C. albicans* colonization in the gut of mice (Matsuo et al., 2019), and serial FMT of *C. albicans* in mice treated with antibiotics results in mutualistic interaction of *C. albicans* and the host: *C. albicans* become a “better” commensal with much lower virulence due to loss-of-function of *FLO8*, which resulted in failure of filamentation and less damage to host cells. The mouse gut-evolved *C. albicans* strains raised trained protective immune responses, and the host was protected against systemic candidiasis (Tso et al., 2018).

Phages account for approximately 90% of the human virome. They can modulate the composition the microbiota at least in part due to their antimicrobial activity (Scarpellini et al., 2015). Predatory bacteria can eat other bacteria or yeasts by actively hunting and consuming their biomass as nutrients (Rossen et al., 2015). In the gut where Gram-negative bacteria predominates, predatory bacteria can be used to restore gut dysbiosis in which Gram-negative bacteria predominates (Atterbury et al., 2011).

## Conclusion

In recent years, due to the treatments for organ transplantations, malignant diseases and HIV/AIDS, and the advances in intensive care unit (ICU) interventions, opportunistic infections are increasing. *Candida* spp. are among the leading pathogens (Pfaller and Diekema, 2007). Colonization is a prerequisite for subsequent infection. *Candida* spp. is a commensal organism in up to 60% of healthy individuals (Kullberg and Arendrup, 2015; McCarty and Pappas, 2016). The shift from a commensal to pathogen is regulated by multifactorial mechanisms, mainly the interplay with the host immune defense and with the microbiota. Uncovering the molecular mechanism of the interactions between *C. albicans* and the resident microbiota will largely facilitate the understanding of the etiology and the searching for novel therapeutic options of fungal as well as bacterial infections. Furthermore, the effect of antifungals on the microbiota also deserves more investigations. New approaches such as drug combination therapy against polymicrobial infections need to be developed. Finally, several therapies including probiotics and FMT are under development with the aim of restoring the microbiota equilibrium.

## Author contributions

FY, Y-yJ, and T-hY: conceptualization. HL, M-xM, C-lJ, and Y-bC: writing—original draft preparation. HL, Y-yJ, T-hY, and FY: writing—review and editing. All authors have read and agreed to the published version of the manuscript.
